# Collagenase-2 (matrix metalloproteinase-8) plays a protective role in tongue cancer

**DOI:** 10.1038/sj.bjc.6604239

**Published:** 2008-02-05

**Authors:** J T Korpi, V Kervinen, H Mäklin, A Väänänen, M Lahtinen, E Läärä, A Ristimäki, G Thomas, M Ylipalosaari, P Åström, C Lopez-Otin, T Sorsa, S Kantola, E Pirilä, T Salo

**Affiliations:** 1 Department of Oral and Maxillofacial Surgery, Institute of Dentistry, University of Oulu and Oulu University Hospital, Oulu, Finland; 2 Department of Diagnostics and Oral Medicine, Institute of Dentistry, University of Oulu and Oulu University Hospital, Oulu, Finland; 3 Division of Statistics, Department of Mathematical Sciences, University of Oulu, Oulu, Finland; 4 Department of Pathology, University of Helsinki, Helsinki, Finland; 5 Tumor Biology Laboratory, Cancer Research UK Clinical Centre, Barts and the London School of Medicine and Dentistry, London, UK; 6 Departamento de Bioquimica y Biologia Molecular, Instituto Universitario de Oncologia, Universidad de Oviedo, Oviedo, Spain; 7 Department of Oral and Maxillofacial Diseases, Institute of Dentistry, University of Helsinki and Helsinki University Central Hospital, Helsinki, Finland

**Keywords:** squamous cell carcinoma, tongue, MMP-8, matrix metalloproteinases, oestrogen receptor, oestrogen

## Abstract

Squamous cell carcinoma (SCC) of the tongue is the most common cancer in the oral cavity and has a high mortality rate. A total of 90 mobile tongue SCC samples were analysed for Bryne's malignancy scores, microvascular density, and thickness of the SCC sections. In addition, the staining pattern of cyclooxygenase-2, *α*v*β*6 integrin, the laminin-5 *γ*2-chain, and matrix metalloproteinases (MMPs) -2, -7, -8, -9, -20, and -28 were analysed. The expression of MMP-8 (collagenase-2) was positively associated with improved survival of the patients and the tendency was particularly prominent in females. No sufficient evidence for a correlation with the clinical outcome was found for any other immunohistological marker. To test the protective role of MMP-8 in tongue carcinogenesis, MMP-8 knockout mice were used. MMP-8 deficient female mice developed tongue SCCs at a significantly higher incidence than wild-type mice exposed to carcinogen 4-Nitroquinoline-*N*-oxide. Consistently, oestrogen-induced MMP-8 expression in cultured HSC-3 tongue carcinoma cells, and MMP-8 cleaved oestrogen receptor (ER) α and β. According to these data, we propose that, contrary to the role of most proteases produced by human carcinomas, MMP-8 has a protective, probably oestrogen-related role in the growth of mobile tongue SCCs.

Squamous cell carcinoma (SCC) of the tongue is the most common type of cancer in the oral cavity and its incidence has increased over the past decades worldwide (Swango, 1996; Alho *et al*, 1999). SCCs of the tongue behave aggressively and almost half of the patients still die of the disease (Dickman *et al*, 1999). Efficient intervention of tongue SCCs requires the identification of the most aggressively behaving tumours. Over the past few years, a series of studies have tried to define immunoreactive molecular markers with ability to predict disease recurrence and prognosis of oral SCC patients (Bettendorf *et al*, 2004; Brinkman and Wong, 2006). In this work, we have addressed this question by analysing at both clinical and molecular levels, a series of tongue cancer cases. This population-based study covered all the patients in Northern Finland with surgically treated SCC in the mobile tongue between the years 1981–2001. Resection samples from 90 eligible mobile tongue SCC patients (47 males, 43 females) were analysed using a set of possible new and previously proposed immunohistochemical prognostic markers including microvessel density (CD31 and factor VIII), cyclooxygenase-2 (COX-2), the laminin-5 *γ*2-chain, integrin *α*v*β*6 and matrix metalloproteinases (MMP-2, -7, -8, -9, -20, and -28). The high expression levels of COX-2 are known to be associated with poor disease-free survival in oral squamous cell carcinoma (Pannone *et al*, 2007) and also laminin-5 *γ*2-chain, a basement membrane protein (Gasparoni *et al*, 2007), and *α*v*β*6 integrin, a transmembrane adhesion protein (Hynes, 1992) are known to be increased in invasive SCCs (Breuss *et al*, 1995; Jones *et al*, 1997; Ramos *et al*, 1997). Angiogenesis is crucial for tumour growth (Folkman *et al*, 1963) and metastasis (Folkman, 1990) but the role of angiogenesis is still controversial in oral SCC (Gasparini *et al*, 1993; Leedy *et al*, 1994; Hogmo *et al*, 1999; Macluskey *et al*, 2000) and thus deserves further investigation. MMPs cleave almost all extracellular matrix proteins but differ in substrate specificity. These enzymes can be divided into the collagenases (MMP-1, -8 and -13), gelatinases (MMP-2 and -9), stromelysins (MMP-3, -10 and -11), matrilysins (MMP-7 and -26), membrane-type MMPs and other MMPs (Overall and Lopez-Otin, 2002). Previous studies have shown inconsistent results with the expression of various MMPs and the outcome of the patients with oral carcinomas. However, in virtually all these previous studies, a heterogenous set of tumours have been analysed and therefore the results may not be comparable (Guttman *et al*, 2004; Ruokolainen *et al*, 2004). Identification of any of these factors as a potential prognostic marker would be useful for estimating the behaviour of the tumour and the survival of the patient with SCC in the mobile tongue. In this work, we provide evidence that collagenase-2 (MMP-8) is a protective factor in mobile tongue SCC. Furthermore, and by using mice deficient in this metalloproteinase and oral carcinoma cells producing MMP-8, we examine putative molecular and cellular mechanisms underlying the protective effect of this enzyme in tongue cancer.

## MATERIALS AND METHODS

### Patients

All 90 patients treated surgically (with at least 5 mm histological margins) at the Oulu University hospital with diagnosed mobile tongue SCC during the years 1981–2001 were included in this study. This tertiary care centre provides primary treatment for all cancer patients in the two northernmost provinces of Finland, covering a population of ∼650 000 inhabitants. The patients (47 males, 43 females) were 26–99 years of age at the diagnosis of SCC (median 62 years). The following tumour-related factors were collected from the hospital files: tumour grade according to the classification (Pindborg *et al* 1987) and TNM stage of the tumour (International Union Against Cancer, 2002). The patient's present status (alive or dead, and the cause of death) was confirmed from the death statistics of the statistics of Finland. Completely resected carcinoma specimens were routinely fixed in 10% formalin, paraffin-embedded and stained with hematoxylin–eosin (HE) for histopathological diagnoses. The study was approved by the Ethical Committee of the Faculty of Medicine, University of Oulu.

### Animals

MMP-8 knockout mice were generated by gene targeting as previously described (Balbin *et al*, 2003). Wild-type mice (C57BL/6) with a similar genetic background were used as controls. All experiments were approved by and performed according to the recommendations of the Animal Care and Use Committee at the University of Oulu.

### Induction of tongue SCC in MMP-8 KO mice

Forty-seven 13–16-week-old mice were exposed to tongue SCC-inducing 4-Nitroquinoline-*N*-oxide (4NQO, Sigma, USA) (Steidler and Reade, 1984; Gannot *et al*, 2004) dissolved in propylene glycol to a final concentration of 10 mg ml^−1^. The mice were lightly anaesthetised by Isofluran (Forene®, Abbott Scandinavia, Sweden) inhalation and 4NQO was smeared to the left dorsal half of the tongue 3 times per week for 12 weeks. The mice were killed at 55 weeks by cervical dislocation after inhalation of CO_2_. The tongues were dissected and fixed routinely in 10% formalin for paraffin embedding. Tissue sections were stained with hematoxylin–eosin. Lesions differentiation was analysed and graded into three classes, (1); normal epithelium without any carcinogenic changes (no change), (2); various (mild, moderate, severe) epithelial dysplasias (dysplasia, including four dysplastic papillomas), and (3); SCC (cancer). Histological diagnoses were done for a total of 47 tongues by an expert pathologist.

### Cell culture

Human oral SCC C1-cells (Ylipalosaari *et al*, 2005) were grown in keratinocyte growth medium including α medium (Gibco BRL, Life Technologies Inc., Grand Island, NY, USA) containing 10% fetal bovine serum (EuroClone, Milano, Italy) and supplemented with antibiotics, 1.8 × 10^−4^ M adenine, 5 *μ*g ml^−1^ insulin, 1 × 10^−10^ M cholera toxin, 0.5 *μ*g ml^−1^ hydrocortisone, 10 ng ml^−1^ epidermal growth factor (all from Sigma Chemical Co., St Louis, MO, USA) and 7.5% sodium bicarbonate. HSC-3 tongue carcinoma cells were grown as previously described (Moilanen *et al*, 2003). HSC-3 cells were maintained in serum-free medium supplemented with 0.5% lactalbumin overnight prior to addition of 10 nM oestrogen (1, 3, 5 (10)-estradien-3 17 *β*-diol Steraloids Inc.) overnight. Conditioned media was collected and the cells were rinsed three times with PBS. The cells were incubated on ice for 10 min with 2% Triton X-100 in PBS for sampling of cell membrane extract. Total proteins were extracted with Trizol® (Gibco BRL, Life Technologies Inc., Roskilde, Denmark).

### RT–PCR

Total RNA was isolated from HSC-3 cells incubated with or without 10 nM oestrogen (1, 3, 5 (10)-estradien-3 17 *β*-diol Steraloids Inc.) using Trizol® (Gibco BRL). RT–PCR was performed as previously described (Moilanen *et al*, 2002) using random decamers (Ambion Europe Ltd., Cambridgeshire, UK). PCR was performed as previously described using MMP-8 primers producing a band of 352 bp (Moilanen *et al*, 2002) and *β*-actin primers purchased from Ambion Europe Ltd. PCR products were analysed by standard agarose gel electrophoresis.

### Antibodies

The following polyclonal antibodies were used in the immunohistochemical stainings: laminin-5 *γ*2-chain (1 : 1000) (Pyke *et al*, 1995), MMP-8 (1 : 200) (Hanemaaijer *et al*, 1997), MMP-9 (1 : 1000, Neomarkers, Fremont, CA, USA), MMP-20 (1 : 1000) (Väänänen *et al*, 2001), MMP-28 (1 : 500) (Lohi *et al*, 2001) and factor VIII (1 : 800, Dako A/S, Glostrup, Denmark). MMP-2 (1 : 2000, Suomen Bioanalytiikka, SBA, Turku, Finland), MMP-7 (1 : 80, Calbiochem, La Jolla, CA, USA), integrin *α*v*β*6 (6.2G2 at 0.5 *μ*g ml^−1^ (Weinreb *et al*, 2004), Biogen Idel, Inc., Cambridge, MA, USA), COX-2 (1 *μ*g ml^−1^, Cayman Chemical, Ann Arbor, Michigan, USA) and CD31 (1 : 30, DakoCytomation, Glostrup, Denmark) antibodies were monoclonal. ER-*α* (1 : 100, MC-20, Santa Cruz Biotechnology Inc., CA, USA) and ER-*β* (1 : 500, Ab-24, Lab Vision, CA, USA) antibodies were polyclonal.

### Immunohistochemical staining

Immunohistochemical staining was done as previously described (Ylipalosaari *et al*, 2005). Briefly, paraffin sections were deparaffinised and pretreated. Sections were incubated with the primary antibody overnight at 4°C. Sections incubated with non-immune rabbit (polyclonal) or non-immune mouse (monoclonal) IgGs instead of primary antibodies were used as negative controls. The sections were incubated with biotinylated secondary antibodies and thereafter with Vectastain Elite ABC reagent (Vector Laboratories, Burlingame, CA, USA). Finally tissue sections were stained with diaminobenzidine (Sigma-Aldrich, St Louis, MO, USA) or 3-amino-9-ethylcarbazole (Zymed, San Francisco, CA, USA) for 10 min. The sections were counterstained with Mayer's haematoxylin (Histolab Products AB, Göteborg, Sweden). C1 carcinoma cells were doublestained with actin and MMP-8 using a method described previously (Ylipalosaari *et al*, 2005). Bound MMP-8 antibody was visualised with Alexa 488-conjugated anti-rabbit secondary antibody (1 : 200 dilution, Invitrogen Ltd., Paisley, UK) and actin was visualised with TRITC-conjugated phalloidin (5 ng ml^−1^, Sigma).

### *In vitro* ER-*α* and ER-*β* cleavage assay

Human recombinant MMP-8 (Chemicon International Inc., Temecula, CA, USA) was tested for the ability to digest human recombinant oestrogen receptor-*α* (ER-*α*) and oestrogen receptor-*β* (ER-*β*) (Invitrogen, CA, USA). In all 3.1 *μ*g of human recombinant ER-*α* and 4.1 *μ*g of human recombinant ER-*β* were used in the assays. The tested enzyme/substrate (E : S) molar ratios were 1 : 11 and 1 : 27 for ER-*β* and 1 : 5, 1 : 12, and 1 : 33 for ER-*α*. The reactions were performed in an incubation buffer (10 mM HEPES, 0.15 M NaCl, 5 mM CaCl_2_ (pH 7.4)) in the presence or absence of MMP inhibitor GM6001, 10 *μ*M (Ryss Laboratories) at 37°C for 22 h. The reactions were stopped by boiling in 4 × electrophoresis sample buffer (250 mM Tris-HCL; 8% SDS; 40% glycerol; 0.0098% Bromphenol blue (pH 6.8)) for 4 min. The samples were then subjected to SDS–PAGE and cleavage products were separated in non-reducing conditions. ER-*α* and ER-*β* were detected by immunoblotting as described.

### Western blotting

Serum-free HSC-3 culture medium was concentrated with 10 K centrifugal filter tubes (Millipore Bedford, MA, USA). The samples were subjected to 10% SDS–PAGE gel electrophoresis and thereafter the proteins were transferred to Immobilon P membrane (Millipore). The membrane was blocked with 5% non-fat milk for 1 h and incubated with MMP-8 antibody (Santa Cruz Biotechnology Inc., CA, USA) at RT overnight. The membrane was washed and incubated with anti-goat secondary antibody (1 : 1000, DAKO A/S, Glostrup, Denmark) for 1 h at RT, washed and incubated with ABComplex/HRP (1 : 1000, DAKO A/S) for 1 h. The membrane was treated with ECL western blotting detection reagent for 1 min and then exposed to Hyperfilm-ECL (Amersham Pharmacia Biotech, Buckinghamshire, UK).

The proteins from the cleavage assays were separated by 12% SDS–PAGE and electrotransferred onto a nitrocellulose membrane (Millipore). To identify the digested fragments, the membranes were incubated overnight with ER-*α* (MC-20, Santa Cruz Biotechnology Inc., CA, USA) antibody against the COOH-terminal part of the receptor at 2 *μ*g ml^−1^ concentration or ER-*β* (Ab-24, Lab Vision, CA, USA) antibody against the COOH-terminal part of the receptor at 2.5 *μ*g ml^−1^ concentration. Biotinylated swine anti-rabbit immunoglobulin G (IgG) secondary antibody (1 : 1000 dilution) (Dako, Glostrup, Denmark) was then allowed to bind for 1 h. Finally the membranes were incubated with avidin/biotinylated horse radish peroxidise (HRP) complex (Dako) for 1 h and the immunoreactive proteins were visualised with ECL western blotting detection reagents (Amersham Biosciences, Piscataway, NJ, USA).

### Malignancy analysis

Hematoxylin–eosin-stained sections were used for Bryne malignancy score analysis (Bryne *et al*, 1992) and for measuring the thickest SCC area. Malignancy score was calculated by determining five morphological features (degree of keratinisation, nuclear polymorphism, number of mitoses, pattern of invasion and lymphoplasmacytic infiltration) from each section and by giving a score (1–4) to each feature. The separate values were then summed up into the final malignancy score (5–20). Samples were divided into two categories: low (5–10) and high (11–20) Bryne score. Thickness of the SCC was determined from the sections by measuring the thickest SCC area with Leica microscope using Leica IM50 Image Manager program. The sections were divided into two groups: those under 6.5 mm and those 6.5 mm or over.

### Immunohistochemical evaluations

All histological evaluations were done at least two times by two to three calibrated investigators without the knowledge of the clinical information of the patients. For microvascular density assessment a method described previously (Weidner *et al*, 1991) was used with slight modifications. Briefly, the most highly vascularised areas (‘hot spots’) based on both factor VIII and CD31 stainings were selected and counted from three different areas: (i) inside carcinoma islands, (ii) carcinoma marginals and (iii) at the edge of the slide, ‘normal looking’ mesenchymal tissue. Cases were divided into three groups: slight, moderate and abundant microvascular density . The levels of COX-2 expression were classified first with low magnification and then with high magnification as follows; grade 0: <1%, grade 2: 1–10%, grade 3: >10–50% and grade 4: >50% of tumour cells. Immunohistochemical staining for the laminin-5 *γ*2-chain was evaluated by dividing each slide into negative (N; no staining within cancer cells), positive (P; cytoplasmic staining within cancer cells) and basement membrane (BM; the tumour nest periphery was partly or circumferentially stained) areas. Parts of different areas were categorised as follows: 1: <20%, 2: 20–40%, 3: 40–60%, 4: 60–80% and 5: 80–100% of the analysed tumour areas. In addition N and P areas separately were analysed with high magnification as follows: 1: <25%, 2: 25–50%, 3: 50–75%, and 4: 75–100% of all tumour cells. The levels of *α*v*β*6-integrin expression were classified as follows: 0=no positive staining, 1=slight positive staining, 2=medium positive staining and 3=strong positive staining in carcinoma cells. The percentage of positively stained cells of the tumour was categorised as follows: 0: <1%, 1: 1–25%, 2: 26–50%, 3: 51–75% and 4: 76–100%. Finally the score (0–7) was calculated by summarising the value of intensity and the category of positively stained cells. Immunostainings for MMPs were evaluated using MMP-2, MMP-7, MMP-8, MMP-9, MMP-20 and MMP-28 antibodies, respectively. The staining intensity and the proportion of the positively stained cells were quantified using a method described previously (Bachmeier *et al*, 2000). Briefly, a five-step grading score was used for the proportion of positively stained carcinoma cells as follows; score 0: <1 cell, score 1: 1 to <25 cells, score 2: 25 to <50 cells, score 3: 50 to <75 cells and score 4:>75 cells. A four-step grading was used for the staining intensity of carcinoma cells as follows; score 0: no positive staining, score 1: weak positive staining, score 2: moderate staining, score 3: strong staining intensity. With MMP-8 and MMP-9 antibodies positively stained cancer cells and positively stained inflammatory cells surrounding the carcinoma islands were counted separately, whereas with MMP-2 and MMP-7 antibodies only positively stained cancer cells were counted. For inflammatory cells only the amount of stained cells was counted, that is, no grading score was used. MMP-20 was excluded from the analysis due to the small number of stained carcinoma cells. The score was calculated by multiplying the mean value of positively stained cells and the mean value of staining intensity. The levels of ER-*β* expression were classified as follows: 0=no positive staining, 1=slight positive staining, 2=medium positive staining and 3=strong positive staining in carcinoma cells and also in inflammatory cells.

### Statistical analysis

Five-year mortalities from SCC itself (with 95% confidence intervals, CI) in various subgroups were estimated by the Kaplan–Meier method. The relative hazards of death from SCC (and 95% CIs) associated with each marker under study were estimated by the Cox proportional hazards regression model, adjusting for the main known prognostic factors (age, sex, and TNM stage of the tumour). Mutual bivariate associations between the various markers were evaluated by computing odds ratios (OR with 95% CIs) for pairs of the dichotomised versions of these variables. The response variable in the mice experiment had three ordered categories: no change, dysplasia, and cancer, but it was dichotomised by pooling dysplasias and cancer into one category. The differences in proportion of developing dysplasia or cancer between the MMP-8 knockout mice and the wild-type C57BL/6 mice, were estimated separately for males and females. This analysis was performed using the function twoby2 in the package Epi, version 0.7.0 (Carstensen *et al*, 2007) attached with the R environment for statistical computing and graphics, version 2.6.0 (R Development Core Team, 2007). All the other statistical analyses were performed using the SPSS software version 12.0.1.

## RESULTS

The overall mortality from the tongue cancer up to 5 years following the diagnosis of the SCC was 23%. Case fatality was generally higher among older patients, those with a more advanced clinical stage, and/or with a higher than average Bryne malignancy score, but thickness of the tumour did not predict the outcome (Table 1). There was no evidence for microvascular density or expression of factors such as COX-2, laminin-5 *γ*2-chain and *α*v*β*6-integrin (Table 2) as being associated with the prognosis. By contrast, it appeared that subjects with positive immunostaining for MMP-2, -8, -9, or -28 in the cancer cells or MMP-9 or ER-*β* in inflammatory cells would have a better prognosis than other patients (Table 3). However, the statistical evidence in support of these observed contrasts was weak.

Bivariate associations between the various markers were also analysed. High Bryne malignancy score value predicted high level of *α*v*β*6-integrin (OR 2.9, 95% CI 1.1–8.2), but MMP-9 and COX2 levels in carcinoma cells (OR 0.17, 95% CI 0.03–0.78; and OR 0.19, 95% CI 0.06–0.65, respectively) were negatively associated with high Bryne category. Likewise, high level of COX-2 predicted high MMP-9 level in carcinoma cells (OR 2.7, 95% CI 1.0–7.3), but laminin-5 *γ*2-chain staining within carcinoma cells was inversely associated with MMP-9 expression (OR 0.22, 95% CI 0.08–0.61).

**Figure 1:**
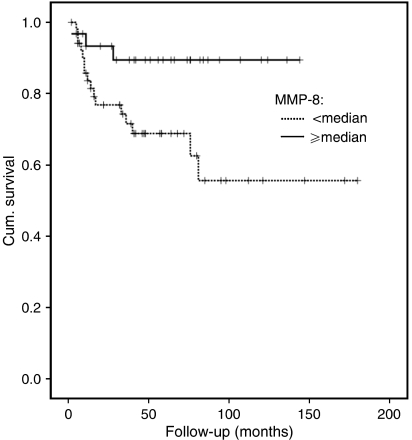
MMP-8 expression in carcinoma cells correlates with improved survival of tongue cancer analysed with the Kaplan–Meier method.

Further analysis revealed that the only statistically significant marker for case fatality was MMP-8 (Table 3). Patients with tumours lacking MMP-8 expression in cancer cells had a relative SCC mortality rate of 3.70 (95% CI 1.04–12.5) compared to patients with some MMP-8 positive immunostaining, when adjusted for age, sex, and stage of tumour (TNM) by the proportional hazards model. MMP-8 or MMP-9 expression in inflammatory cells was not associated with survival (Table 3). In addition, for all evaluated factors only MMP-8 came up with Cox's regression model when age, gender and stage were installed as main variables. Interestingly, positive MMP-8 expression and improved survival also showed a tendency to be more prominent in female tongue cancer patients than in male tongue cancer patients, but this difference was not found to be statistically significant. The mortality of patients with no positive MMP-8 immunostaining also increased over time during the 5-year period (Figure 1). The associations of MMP-8 with gender, oestrogen receptor levels (separately in cancer cells and in inflammatory cells), tumour thickness, TNM stage, and tumour grading were not statistically significant.

**Figure 2:**
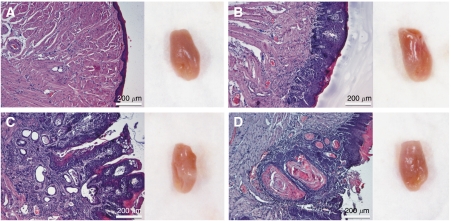
Histopathological and clinical analyses of 4NQO-treated tongues from MMP-8 KO and C57BL/6 mice. (**A**) Normal C57BL/6 male mouse mucosa stained with hematoxylin and eosin. Clinical tongue on the right. (**B**) MMP-8 KO male with dysplasia. (**C**) MMP-8 KO females with invasive SCC. (**D**) MMP-8 KO females with invasive SCC. Scale bar=200 *μ*m.

To test the hypothesis that MMP-8 plays a protective role in tongue SCC, 23 MMP-8 knockout (KO) and 24 wild-type C57BL/6 mice were subjected to chemical carcinogenesis with 4-Nitroquinoline-*N*-oxide (4NQO) for 12 weeks. Half (6/12) of the MMP-8 KO female mice in contrast to none of the 12 wild-type C57BL/6 female mice developed tongue cancer during the experiment (Table 4, Figure 2). Similarly, dysplasias were more frequent in the tongues of MMP-8 KO female mice (4/12) than in wild-type mice (2/12). In male mice no difference in carcinoma development was found between the mouse groups. There was a strong statistical support to the observation that the MMP-8 KO female mice developed tongue cancer more often than the wild-type mice. The estimated proportion of female MMP-8 KO mice developing either dysplasia or carcinoma was 67 percent points higher than that of the female wild-type mice (83 *vs* 17%; 95% CI for the difference in proportions: +21 to +85 percent points). In male mice the same contrast was observed to be 20 percent points (95% CI −21 to +55 percent points).

**Figure 3:**
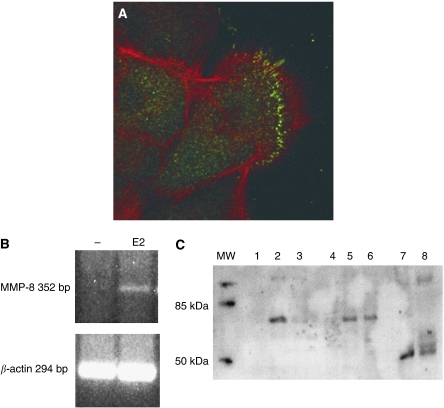
Localisation and molecular forms of MMP-8 in cultured oral carcinoma cells. Localisation of MMP-8 immunoreactivity in oral SCC cell membranes and in intracellular granules is demonstrated in green and red staining demonstrates actin (**A**). MMP-8 and *β*-actin RT–PCR from HSC-3 tongue carcinoma cells incubated with or without 10 nM oestrogen (E2) overnight (**B**). Molecular sizes of MMP-8 in HSC-3 tongue carcinoma cells analysed by western blotting (**C**). Lanes 1 (no E2) and 4 (E2 added) represent concentrated medium where no MMP-8 is detected. Lanes 2 (no E2) and 5 (E2 added) represent the cell membrane extracts where a 75 kDa species can be seen. Lanes 3 (no E2) and 6 (E2 added) are total proteins where a 75 kDa species is visible only after E2 treatment. Lane 7 is odontoblast medium and lane 8 human saliva used as positive controls where a 58 kDa form of MMP-8 can be detected.

MMP-8 expression was further analysed in cultured oral SCC cells by confocal immunofluorescence, which localised MMP-8 immunoreactivity mainly to the tongue carcinoma cell membranes and to subcellular granules. Lack of MMP-8 was found to have different effect on male and female mice when experimental SCC was induced. We therefore investigated the effect of oestrogen on HSC-3 cells. Molecular forms of MMP-8 were identified from HSC-3 tongue carcinoma cells by using western blotting which demonstrated a 75 kDa species in HSC-3 cell membrane extracts. Oestrogen treatment induced expression of a 75 kDa MMP-8 species in HSC-3 cells as evidenced by western blotting. To verify this observation we also performed MMP-8 RT–PCR from HSC-3 cells incubated with or without 10 nM oestrogen overnight and found that MMP-8 mRNA was undetectable in resting HSC-3 cells while oestrogen treatment induced MMP-8 mRNA expression (Figure 3).

**Figure 4:**
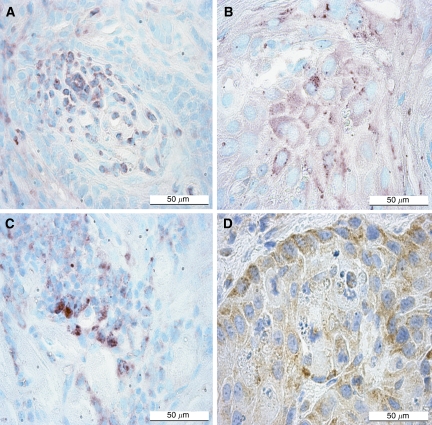
Oestrogen receptor-*α* and -*β* immunohistochemical staining in tongue squamous cell carcinoma. Nuclear and cytoplasmic ER-*α* and ER-*β* positivity (red staining) were detected both in mouse and human tongue SCC cells. (**A**) Mouse SCC stained with ER-*α* antibody (MC-20). (**B**) Human SCC stained with ER-*α* antibody (MC-20) (**C**) Mouse SCC stained with ER-*β* antibody (ab-24). (**D**) Human SCC stained with ER-*β* antibody (ab-24). Scale bars=50 *μ*m.

Because oestrogen acts through the oestrogen receptors, we investigated the expression of ERs in tongue SCCs. Both oestrogen receptor-*α* (ER-*α*) and oestrogen receptor-*β* (ER-*β*) were expressed in human and mouse tongue SCC cells as detected by immunohistochemical methods (Figure 4). ER-*β* expression was found to weakly correlate with a better prognosis (Table 3).

**Figure 5:**
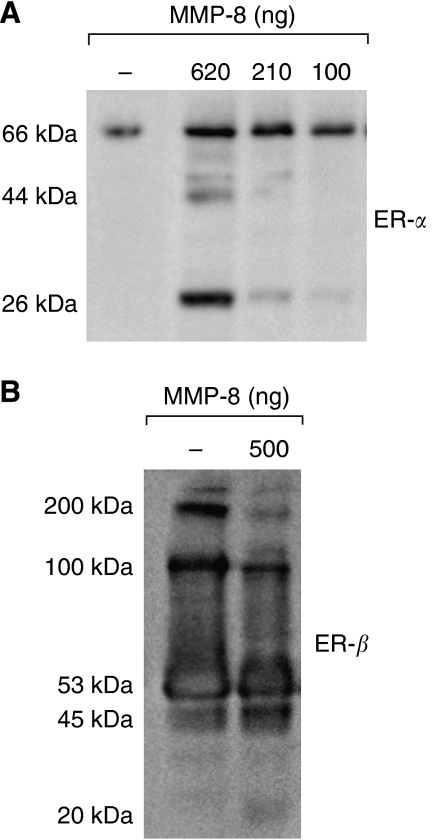
Cleavage of ERs by MMP-8 *in vitro*. Recombinant MMP-8 was incubated with recombinant ER-*α* and ER-*β* at different enzyme-substrate ratios. The cleavage fragments were separated by SDS-PAGE and identified by Western immunoblotting using ER-specific antibodies. (**A**) ER-*α* was incubated with an increasing amount of MMP-8. The cleavage products of approximately 44 and 26 kDa were detected with ER-*α* (MC-20) antibody (**B**). The ER-*β* (ab-24) antibody detected the cleavage products of about 45 and 20 kDa. In addition, the higher molecular weight forms of ER-*β* with approximate sizes of 100 and 200 kDa were diminished after incubation with MMP-8.

To test, whether MMP-8 cleaves ER-*α* or ER-*β*, and thus could have effect on their function, we performed an *in vitro* cleavage assay using purified recombinant MMP-8 and ERs. MMP-8 was found to cleave ER-*α in vitro* dose dependently (Figure 5). Two cleavage products of full length ER-*α* (66 kDa) were detected, with the approximate molecular weights of 44 and 26 kDa by western immunoblotting with an ER-*α* antibody. The intermediate cleavage product of 44 kDa was detected only with the enzyme/substrate molar ratio 1 : 5 (Figure 5A). Only minor cleavage of ER-*β* by MMP-8 could be detected (Figure 5B, only the result from E : S ratios 1 : 11 shown). Approximately 20 kDa and 45 kDa cleavage products of monomeric 53 kDa ER-*β* increased, and around 100 kDa dimeric and 200 kDa higher molecular weight forms of ER-*β* slightly diminished after incubating with MMP-8 (Figure 5B). The broad-spectrum MMP inhibitor GM6001 abolished the ERs degradation by MMP-8 (not shown).

## DISCUSSION

Our investigation was based upon a unique population-based collection of 90 surgically treated SCC resection samples of the mobile tongue. Treatment of the patients in Northern Finland is very effective; the overall mortality from the disease up to 5 years was only 23% in this study, while it generally varies within about 40–55% in Finland (Dickman *et al*, 1999). Worldwide, head and neck cancer is the sixth most common cancer (Hunter *et al*, 2005). Low mortality in this case may hide the significance of some individual prognostic markers, since patients outcome is most likely affected more by the effective diagnostic and treatment strategies than by any of the biological variables. The prognosis was significantly better in younger patients (26–70 years), in patients with lower clinical stages of the tumours (TNM I and II) and in patients with lower (5–10) Bryne's malignancy score as has been shown earlier by us (Kantola *et al*, 2000) and other investigators (Piffko *et al*, 1997). Our results were in line with Teixeira *et al* (1996), who also did not find a correlation between the thickness of the cancer in the deepest tumour areas and outcome of the patients, but in contrast with a study by Charoenrat *et al* (2003) where tumour thickness and survival of the tongue SCC patients correlated significantly. Also in line with previous tongue SCC studies, we did not observe any association between micro vessel density either within the borders or outside the invasive SCC tissue and prognosis of the patients (Leedy *et al*, 1994; Hogmo *et al*, 1999). Unlike Pannone *et al* (2007), we could not find a correlation between COX-2 expression and survival, but COX-2 expression was negatively associated with high Bryne's category, which is seen as a predictor for poor prognosis (Bryne *et al*, 1992). Unlike published earlier (Ono *et al*, 1999; Katoh *et al*, 2002; Gasparoni *et al*, 2007), cytoplasmic or basement membrane zone staining for the laminin-5 *γ*2-chain in cancer cells did not correlate with the outcome of the patient in our study. There are several reports showing increased *α*v*β*6 integrin expression in invasive SCCs (Breuss *et al*, 1995; Jones *et al*, 1997; Ramos *et al*, 1997), but these findings could not be replicated here, although our results, suffering from wide error margins, were not in any conflict with the previous ones. Over 90% of our tongue cancers samples did express *α*v*β*6 integrin but the expression level did not statistically correlate with the outcome of the patients. However, high level of *α*v*β*6 integrin was associated with high Bryne malignancy score category and may thus be linked to more aggressive behaviour and poorer prognosis of the cancer.

MMPs are known to be overexpressed in pathological stages requiring matrix turnover (Overall and Lopez-Otin, 2002). Of the molecules analysed in this study, the statistical evidence was insufficient to make judgments on the association between the expression of MMP-2, -7, -9, and -28 with survival from SCC of the tongue. In previous studies, MMP-9 expression is associated with poor prognosis in tongue and also head and neck SCCs (Juarez *et al*, 1993; Kawamata *et al*, 1998; Nyberg *et al*, 2002) but there is also publications where such a correlation has not been found Kim *et al*, 2006) However, in our bivariate analysis we observed a positive association between MMP-9 and COX-2 levels in carcinoma cells, but negative association with MMP-9 presence and high Bryne category suggesting that MMP-9 in these samples reflects, but not statistically significantly, better prognosis of the disease. In addition, our MMP-28 findings were in the line with Lin *et al* (2006). We found a slight trend between positive MMP-28 immunostaining and better prognosis of tongue squamous cell carcinoma. By contrast, parallel analysis with MMP-8 revealed that production of this protease was significantly associated with good clinical outcome in tongue cancer patients. Decock *et al* (2007) showed that genetic variation in the MMP-8 gene could influence breast cancer prognosis. They also noticed that MMP-8 inhibited breast cancer metastasis. These observations are also in the line with previous studies where MMP-8 expression was associated with a better prognosis of skin cancer in mice (Balbin *et al*, 2003) and a benign behaviour of cultured breast cancer cells (Agarwal *et al*, 2003). However, Stadlmann *et al* (2003) found that MMP-8 expression in ovarian cancer was associated with a poorer prognosis. This is probably due to the fact that different cancer types, or species for that matter, cannot always be directly compared with each other. This is the first study to show that MMP-8 expression in human tongue cancer is significantly linked with prolonged survival. Consistent with this, we also observed that MMP-8 deficient mice were more susceptible to tongue SCC than control mice, this contrast appearing to be more pronounced in females than in male mice. In addition, we found that MMP-8 is cell-membrane associated in cultured tongue carcinoma cells and that oestrogen can induce MMP-8 mRNA and protein expression in HSC-3 tongue carcinoma cells which could partially explain the differences in cancer susceptibility between the genders. Similarly, oestrogen-induced MMP-26 expression has been associated with improved survival in breast cancer (Savinov *et al*, 2006). MMP-8 expression was previously linked to positive characteristics in cultured breast cancer cells (Agarwal *et al*, 2003) and in mouse cancer studies (Balbin *et al*, 2003). Unlike the MMP-26 gene promoter, the MMP-8 gene promoter does not include an oestrogen receptor element. In contrast, it includes the CCAAT/enhancer-binding protein (C/EBP) element (Khanna–Gupta *et al*, 2005). C/EBP has been shown to associate with ER-*α* and the complex then acts as a transcription factor and can regulate gene promoter activity (Chen *et al*, 2006). This could explain why oestrogen induces MMP-8 production in HSC-3 cells. Li *et al*, 2004 found the MMP-26 gene promoter activity to be stimulated by oestrogen through the ERs. In addition, MMP-26 can cleave ER-*β in vitro* and plays an important antitumorigenic role in hormone-regulated malignancies by regulating the amount of ER-*β* and thus regulating the oestrogen signalling pathway (Savinov *et al*, 2006). In this study we found, for the first time, that although ER-*β*, but not ER-*α*, are expressed in normal oral mucosa (Välimaa *et al*, 2004) they were both faintly produced in the tongue SCC islands. ER-*β* is expressed by lymphocytes (Törnwall *et al*, 1999; Ulziibat *et al*, 2006), and interestingly we found a weak correlation for the expression of ER-*β* in inflammatory cells with prolonged survival. We also found that MMP-8 can effectively cleave ER-*α* and some cleavage of ER-*β in vitro* was also detected. Additionally, a dimeric (about 100 kDa) and a higher molecular weight (around 200 kDa) form of ER-*β* monomer (53 kDa) diminished after incubating with MMP-8. According to these data, it is possible that MMP-8 cleaves the ER-*β* dimer and complex forms also *in vivo*. Dimerisation and stability of ER dimers is crucial for oestrogen receptor activation and function as a transcription factor (Tamrazi *et al*, 2002). These data suggest that MMP-8 protective role could be related to its ability to regulate the amount of ERs and thus regulating oestrogen-signalling pathway during tumour development, especially in hormone-regulated malignancies. This finding also at least partly explains the differences between MMP-8 KO male and female mice cancer susceptibility. Our study now supports these data also in human patients with mobile tongue SCC, the most common type of oral cancer.

In conclusion, based on both human SCC tissue sample analysis and mice *in vivo* carcinogenesis experiments, our study is the first to provide evidence that carcinoma cell membrane bound MMP-8 should be considered as a protective anti-tumour factor in mobile tongue SCC and its mechanism of action in tumours may be oestrogen related.

## Figures and Tables

**Table 1:** The disease-specific five-year mortality from 90 tongue SCC patients

**Factors**	***n***	**5-year mortality (%)**	**95% CI**
*Gender*			
Female	47	22	9–35
Male	43	25	10–40
			
*Age group*			
26–70 years	59	18	7–29
>70 years	31	35	15–55
			
*Clinical stage* a			
I and II	51	14	3–25
III and IV	36	32	15–49
			
*Malignancy score (Bryne)* a			
Low score	22	5	0–14
High score	65	31	18–44
			
*Thickness of SCC*			
<6.5 mm	33	25	8–42
⩾6.5 mm	57	27	15–39

a The clinical stage was not reported and the malignancy score not analysed from three patients.

**Table 2:** The disease-specific five-year mortality from 80–84 tongue SCC patients for histological prognostic factors

**Factors**	***n***	**5-year mortality (%)**	**95% CI**
*Microvessel density*			
Inside carcinoma islands			
Slight	26	25	5–45
Moderate or abundant	58	25	13–37
Carcinoma marginals			
Slight	29	27	7–47
Moderate or abundant	54	24	12–36
‘Normal looking’ mesenchymal tissue			
Slight	36	30	14–46
Moderate or abundant	47	20	7–33
			
*Expression of COX-2*			
Overview ( × 100 magnification)			
<1% positively stained cells	40	25	10–40
1–50% positively stained cells	44	21	7–35
Detailed view from ‘hot spots’ ( × 400 magnification)			
<1% positively stained cells	25	28	8–48
1–10% positively stained cells	28	14	1–27
>10% positively stained cells	31	29	10–48
			
*Expression of laminin-5 γ2-chain*			
Overview ( × 100 magnification)			
No staining in cancer cells			
<40% of the tumour area	63	19	8–30
⩾40% of the tumour area	20	31	10–52
Cytoplasmic staining in cancer cells			
<40% of the tumour area	57	17	6–28
⩾40% of the tumour area	26	36	13–59
Staining in basement membrane			
<40% of the tumour area	23	27	6–48
⩾40% of the tumour area	60	21	10–32
Detailed view from ‘hot spots’ ( × 400 magnification)			
Negatively stained cells			
<50% of all cancer cells	23	17	0–35
⩾50% of all cancer cells	60	24	12–36
Positively stained cells			
<50% of all cancer cells	10	12	0–36
⩾50% of all cancer cells	73	23	12–34
			
*Expression of αvβ6-integrin (score 0–7)*			
0	7	31	0–69
3–5	28	12	0–25
6–7	45	29	14–44

**Table 3:** The disease-specific five-year mortality from 80–84 tongue cancer for histological prognostic factors

**Factors**	***n***	**5-year mortality (%)**	**95% CI**
*MMP-2 in cancer cells, score*			
<0.32	54	28	15–41
⩾0.32	30	15	1–29
			
*MMP-7 in cancer cells, score*			
<1.84	52	24	11–37
⩾1.84	21	26	6–46
			
*MMP-8 in cancer cells, score*			
<0.78	52	31	17–45
⩾0.78	31	11	0–23
			
*MMP-8 in inflammatory cells*			
<0.08	42	24	10–38
⩾0.08	41	24	10–38
			
*MMP-9 in cancer cells, score*			
<2.62	53	29	15–43
⩾2.62	30	19	4–34
			
*MMP-9 in inflammatory cells*			
<10.20	41	32	16–48
⩾10.20	42	18	5–31
			
*MMP-28 in cancer cells, score*			
<0.09	58	26	13–39
⩾0.09	24	19	2–36
			
*ER-β in cancer cells, staining intensity*			
0–1=No or slight positive staining	31	19	4–35
2–3=Medium or strong positive staining	35	29	12–45
			
*ER-β in inflammatory cells, staining intensity*			
0–1=No or slight positive staining	34	25	8–41
2–3=Medium or strong positive staining	33	23	8–38

**Table 4:** Histological incidence of lesions in 4NQO-treated tongue sections from MMP-8 KO and wild type mice

**Response**					
**Sex**	**Strain**	**No change**	**Dysplasia**	**Carcinoma**	**Total**
Male	C57BL/6	9	2	1	12
	MMP-8 KO	6	4	1	11
Female	C57BL/6	10	2	0	12
	MMP-8 KO	2	4	6	12
